# Secondary neurulation mechanisms likely arose in vertebrates during mesenchymal tailbud extension

**DOI:** 10.3389/fcell.2026.1870085

**Published:** 2026-09-25

**Authors:** Daiki Sugita, Alexis Lauga, Martin Minařík, Lucy Barlow, Nicole Campbell, Michaela Vazačová, Martin Pšenička, Roman Franěk, Clare Buckley

**Affiliations:** 1 Department of Physiology, Development and Neuroscience, University of Cambridge, Cambridge, United Kingdom; 2 School of Clinical Medicine, University of Cambridge, Cambridge, United Kingdom; 3 Department of Zoology, Charles University, Faculty of Science, Prague, Czechia; 4 Division of Molecular and Cellular Function, Faculty of Medicine, Biology and Health, University of Manchester, Manchester, United Kingdom; 5 FMBH Histology Core Facility, University of Manchester, Manchester, United Kingdom; 6 Faculty of Fisheries and Protection of Waters, South Bohemian Research Center of Aquaculture and Biodiversity of Hydrocenoses, University of South Bohemia in Ceske Budejovice, Vodnany, Czechia

**Keywords:** neural tube, secondary neurulation, sterlet, tailbud extension, vertebrate evolution

## Abstract

**Introduction:**

The central nervous system (CNS) is an essential organ system common to all vertebrates and is one of the defining features of the Chordata phylum. In Chordates, neural tubes may form via either primary neurulation, secondary neurulation, or a mixture of the two. During primary neurulation, there is folding of an already-polarized epithelium. In contrast, secondary neurulation involves mesenchymal to epithelial transition, cell interdigitation and sometimes *de novo* cavitation from an initially solid rod of mesenchymal cells. Current studies only show secondary neurulation in a subset of chordates, and it is unclear why this should be the case.

**Methods:**

This article uses published literature to reevaluate the mode and the relative timings of neurulation and tailbud extension in chordate species. It also uses micro-computerized tomography (microCT) and immunohistochemistry (IHC) to investigate the cellular mechanisms of neurulation within Actinopterygii fish, in which so far only teleosts have been shown to undergo secondary neurulation.

**Results:**

Our literature review suggests that early branching chordates likely neurulate via exclusively primary neurulation and do not have a mesenchymal tailbud. In contrast, in the Vertebrate species in which secondary neurulation has been previously documented, there is a strong positive correlation between the onset of secondary neurulation and of a mesenchymal tailbud extension. The mechanisms of neurulation have not been well characterised across the Chordates, resulting in a patchy reported distribution of secondary neurulation. MicroCT images demonstrated that tropical gar fish from the Holostei clade neurulate via secondary-like mechanisms along most of their axis, whilst Senegal bichir (Cladistia clade) and sterlet sturgeon (Chondrostei clade) exhibit primary neurulation in anterior regions and have an undifferentiated tailbud at the posterior end of the embryo. Higher resolution IHC data demonstrated that the sterlet embryo neural tube forms via primary mechanisms until a neuroenteric canal, whilst proliferative, Sox2-positive cells in the tailbud intercalate into the extending neural tube via secondary mechanisms of neurulation, without *de novo* lumen formation.

**Discussion:**

Together, our findings suggest that the cellular mechanisms of secondary neurulation likely evolved in the Vertebrata subphylum to enable cells from an extending mesenchymal tailbud to be incorporated into an already polarised posterior spinal cord. However, it remains unclear why different cellular mechanisms of secondary neurulation exist between species (e.g. *de novo* polarisation and cell interdigitation vs. only cell interdigitation) and why some species undergo secondary neurulation along the majority of their axis whilst in others it is limited to the tailbud. We suggest that varying geometrical constraints due to differences in relative rates of body axis elongation, mesenchymal cell proliferation and MET are likely key to which specific mechanisms of neurulation are adopted.

## Introduction

Neurulation is a crucial morphogenetic process that generates the neural tube, most of which later develops into the central nervous system. Proper formation of the neural tube is therefore important for vertebrate development, and dysregulation of this morphogenetic event leads to neural tube defects (NTDs), which are among the most common congenital defects in humans (Greene and Copp, 2014; ten Donkelaar et al., 2023).

In amniotes, there are broadly two mechanisms of neurulation: primary and secondary neurulation. Primary neurulation occurs in the brain and trunk region and involves folding of the neural plate into a neural groove, followed by medial movement of neural folds leading to closure of the neural tube (Colas and Schoenwolf, 2001; Shum and Copp, 1996). Defects in the folding and fusion processes during primary neurulation lead to a variety of open NTDs, depending on which axial region is affected (Copp et al., 2015). Secondary neurulation contributes to the neural tube in caudal regions (Catala, 2021; Colas and Schoenwolf, 2001) and involves the mesenchymal-to-epithelial transition (MET) of cells and canalization of an initially solid tailbud. In some species such as humans and mice, the most caudal part of the secondary neural tube later regresses (Santos et al., 2024), but a functional portion remains throughout the life of the animal. The mechanisms of secondary neurulation are much less well understood than those of primary neurulation, and therefore, mechanisms causing defects of the secondary neural tube are also not well understood. However, it is likely that defects in secondary neurulation contribute to spinal dysraphisms such as spinal lipoma, lipomyelomeningocele, and cord tethering (Copp and Greene, 2013; Jones et al., 2019). Theoretically, defects in secondary neurulation mechanisms such as cell ingression could even contribute to some open NTDs (Catala, 2021). Therefore, understanding this process and, importantly, species-specific differences in the mechanisms of secondary neurulation between different model organisms is important to further knowledge of the origins of these diseases and how best to study them experimentally.

In avian species such as chicken, hollowing of the tailbud occurs to form multiple lumens (Schoenwolf and Delongo, 1980), which are resolved into a single lumen via SMAD3-mediated cell intercalation (Gonzalez-Gobartt et al., 2021). However, in mammals such as mice, the primary and secondary neural tubes are always continuous, and the main mechanisms of secondary neurulation are the formation of a medullary rosette and intercalation of cells into the existing tube (Schoenwolf, 1984). Human secondary neurulation has historically been assumed to occur in a similar way to avian embryos, and a high prevalence of neural tube defects was reported to occur at a region of junctional neurulation, where elements of primary and secondary mechanisms are present at the same axial level in the chick (Dady et al., 2014). However, a recent extensive analysis of human embryos has found no evidence for such a transition zone in humans (Santos et al., 2024). While clear evidence of multiple lumens was seen within the tailbud, these did not appear to arise as completely separate structures. Instead, a mechanism somewhere in between the chick and mouse generates multiple lumens that all remained connected to the initial neural tube (Santos et al., 2024). Together, it is clear that, even within the amniotes, there is variation in cellular mechanism. The cellular mechanisms that are conserved within secondary neurulation of all amniotes are MET and cell intercalation.

Non-amniote vertebrate species have a variety of modes of neurulation, sometimes encompassing combined mechanisms of both primary and secondary neurulation along much of the anterior–posterior axis (Araya et al., 2016; Harrington et al., 2009). For example, during *Xenopus laevis* neurulation, the neural plate bends to form a neural groove, mediated by apical constriction of superficial neural cells, leading to neural fold fusion (similar to primary neurulation). After neural fold closure, neurulation continues by medial migration, radial intercalation, and epithelialization of deep neural cells, leading to lumen reconstruction (similar to the intercalation in secondary neurulation) (Davidson and Keller, 1999; Harrington et al., 2009; Schroeder, 1970). Zebrafish neurulation of the hindbrain and spinal cord occurs via early invagination and intercalation of cells (Araya et al., 2019; Papan and Campos-Ortega, 1994), followed by secondary-like *de novo* polarization and lumenogenesis (Buckley et al., 2013; Clarke, 2009; Lowery and Sive, 2004; Schmitz et al., 1993). More anterior brain regions incorporate elements of primary folding mechanisms alongside these secondary-like hollowing mechanisms (MacGowan et al., 2025; Werner et al., 2021). Therefore, the specific combination of neurulation modalities varies, both between species and along the axial axis of individual organisms. While current literature suggests that primary neurulation is an ancestral mode, secondary mechanisms are documented in only a subset of chordates (Handrigan, 2003), while some are suggested to neurulate via exclusively primary mechanisms (Holland, 2015; Nicol and Meinertzhagen, 1988). Together, this raises questions of when secondary neurulation evolved and why.

A defining feature of chordates is the formation of a post-anal tail. Formation of this structure requires significant posterior elongation of axial tissues, incorporating the neural tube, notochord, and paraxial mesoderm (Benazeraf et al., 2017; Benazeraf and Pourquie, 2013; Steventon et al., 2016). One important mechanism of body elongation is convergent extension, during which cell–cell rearrangements cause a narrowing and elongation of tissue (Wallingford et al., 2002). Cell–cell rearrangement is also thought to continue locally during tailbud extension. Recent investigations within the rapidly extending zebrafish tailbud have demonstrated that tail elongation in teleosts is driven by collective tissue flows, which require cells to be in a more mesenchymal state in order to retain fluidity. Cells then transition to a more solid state in the anterior tailbud, promoting posterior axis elongation (Mongera et al., 2018). The source of such mesenchymal tailbud cells, investigated in vertebrate embryos, is thought to be a posteriorly located progenitor zone containing bipotent neuromesodermal progenitors (NMPs), expressing neural progenitor marker Sox2 and early mesodermal marker T/Bra (Henrique et al., 2015). Cell proliferation within the posterior NMP zone helps control the coordinated specification of tail regions into neural and mesodermal tissues. The relative contribution to tailbud extension of posterior volumetric cell proliferation and cell–cell rearrangement versus anterior mechanisms of volumetric growth and convergence extension differs greatly between species (Sambasivan and Steventon, 2020). However, the incorporation of any initially mesenchymal tailbud cells into an already polarized neural tube would require secondary neurulation mechanisms to facilitate cell intercalation into the neuroepithelium. Therefore, a straightforward way to determine whether secondary-like neurulation mechanisms occur in the tail is to test for the presence of a mesenchymal tailbud containing neural progenitor cells that are unpolarized and not yet integrated into the neural tube. There is currently no protein marker that is unique for neural progenitor cells. However, the presence of the neural progenitor marker Sox2 within mesenchymal tailbud cells would demonstrate the presence of either neural progenitor cells or of neuromesodermal progenitor cells. Sox2 expression is downregulated in NMPs as they become mesodermally fated and maintained in NMPs as they become neurally fated (Takemoto et al., 2011). The levels of Sox2 expression can therefore be used to help define the fate of tailbud cells (Morabito et al., 2025).

Based on the variety and overlapping of modes of neurulation, both between species and within individual organisms, we hypothesized that the reported patchy distribution of secondary neurulation mechanisms amongst vertebrate species might reflect the incomplete investigation of neurulation mechanisms across the evolutionary tree, rather than the independent evolution of these mechanisms on multiple occasions. Instead, we hypothesized that secondary neurulation mechanisms arose in a single common ancestor within the chordates in response to the development of mesenchymal tailbud extension. To test this hypothesis, we re-examined current literature to examine coverage of the investigation into the cellular mechanisms of neurulation. We compared the developmental timings of documented secondary neurulation with the onset of posterior tailbud extension. Lastly, we analyzed the morphology of developing neural tubes from the Cladistia, Chondrostei, and Neopterygii chordates within Actinopterygii to investigate the cellular mechanisms of neurulation within this chordate class.

## Materials and methods

### MicroCT scans

MicroCT scans previously used to investigate facial structure development in non-teleost fishes (Minarik et al., 2017) were digitally resliced using the TransformJ Fiji plugin (Erik Meijering) (Schindelin et al., 2012) to investigate the neural tissue structure of three different species from the Actinopterygii class: the Senegal bichir (*Polypterus senegalus*), the sterlet sturgeon (*Acipenser ruthenus*), and the tropical gar *(Atractosteus tropicus*). For each of the species, three developmental stages were obtained to show neurulation processes at both the pre- and post-tailbud stages. For the Senegal bichir (*P. senegalus*), these were ST19, ST22, and ST24. For the sterlet sturgeon (*A. ruthenus*), these were ST22, ST25, and ST27. For the tropical gar *(A. tropicus*), these were ST15, ST17, and ST18.

Samples for micro-computerized tomography (CT) were fixed in 4% PFA and then postfixed in Karnovsky fixative overnight or longer and stored in 70% ethanol. Contrasting of soft tissues was achieved by incubation in 1% phosphotungstic acid (PTA) in 70% ethanol overnight. Contrasted samples were then mounted in pipette tips using 2.5% agar and, once set, imaged using a MicroXCT (Xradia) scanner at 2.0 μm voxel size.

### Sterlet embryo staging and fixation

The staging of sterlet development was based on features of Russian sturgeons *Acipenser gueldenstaedtii* (Dettlaff et al., 1993):

**Table:** 

Stage	Characteristic
S20	Neural plate stage
S25	The rudiment of posterior trunk and tail regions
S26	Onset of a separation of a rudiment of tail regions
S27	Short cardiac tubes, tail elongation

Sterlet embryos were fixed in 4% PFA in 0.1 M PBS for 4 h at room temperature and then rinsed in PBS, dechorionated, and gradually dehydrated to 100% ethanol for shipment. Once received, they were stored at −20 °C in 100% ethanol until embedding.

### Manual processing of embryos

Some sterlet embryos were manually processed at the University of Cambridge, where they were washed with 100% ethanol for 5 min three times and washed in 1:1 ethanol: Histo-Clear II (National Diagnostics, HS-202) for 1 h at room temperature. Embryos were incubated in Histo-Clear II for at least 1.5 h twice, and the second wash was carried out at 70 °C in a wax oven. Then they were washed in 1:1 Histoclear II:Paraplast Plus for at least 1.5 h at 70 °C in a wax oven. The timing of washing began after making sure this mixed solution had completely melted. They were transferred to Paraplast Plus and incubated for at least 1.5 h, and this was repeated several times at 70 °C in a wax oven by replacing the Paraplast Plus. After the final replacement, embryos were incubated overnight at 70 °C in a wax oven.

After incubation with Paraplast Plus overnight, embryos were transferred to a plastic embedding mold and oriented for transverse or sagittal sectioning. Once correctly oriented, the molds were left at room temperature for at least 12 h to let the Paraplast Plus solidify. Embedded embryos were sectioned into 8 µm sections using a microtome. Sections were then placed onto SuperFrost Plus adhesion microscope slides and spread with distilled water, followed by heating at 40 °C for 30 min. The water was carefully removed from the slides, and the slides were dried at 40 °C overnight.

### Automatic processing of embryos

Some sterlet embryos were automatically processed using the Leica SP300 tissue processor in the Bioimaging facility at the University of Manchester. The processing protocol was as follows:

**Table:** 

Reagent	Temperature	Time
70% EtOH	Ambient	5 min
100% EtOH	Ambient	5 min
100% EtOH	Ambient	5 min
100% EtOH	Ambient	5 min
Xylene	Ambient	20 min
Xylene	Ambient	20 min
Xylene	Ambient	30 min
Wax I	65 °C	1 h 10 min
Wax II	65 °C	1 h 10 min
Wax III	65 °C	1 h 20 min

Embryos were then sectioned into 8 µm sections using a Leica Rx multicut microtome and placed onto Superfrost adhesion microscope slides as in Cambridge.

### Immunohistochemistry

The slides were washed with Histo-Clear II for 5 min twice and rehydrated with decreasing concentrations of ethanol (100%, 70%, 50%, and 30%), each step for 5 min. Then the slides were rinsed with distilled water for 2 min twice and with PBS for 5 min three times. The slides were washed with PBT (PBS with 0.1% Triton X-100) for 5 min three times. Heat-induced antigen retrieval was carried out to enable primary antibodies to access the antigens: The slides were warmed in distilled water pre-heated to 70 °C in a wax oven for 5 min and incubated in sodium citrate solution (10 mM sodium citrate, pH 6.0) for 25 min at 95 °C. Then slides were immediately cooled at −20 °C for 30 min. After washing with PBT for 5 min twice, the slides were blocked with a blocking solution for 2 h (10% goat serum in PBT), then incubated in primary antibodies overnight at 4 °C. The primary antibodies used were against Zona occludens 1 (ZO-1, mouse monoclonal, Invitrogen, 33–9100, 1:500), Sox2 (rabbit polyclonal, GeneTex, GTX124477, 1:125), and Proliferating Cell Nuclear Antigen (PCNA, mouse monoclonal, Sigma-Aldrich, P8825, 1:500). The primary antibody solutions were then washed off with PBT for 10 min three times. The slides were blocked for 2 h and incubated overnight with secondary antibodies conjugated to a fluorophore at 1:500 and Hoechst at 1:1000. Donkey anti-mouse Alexa Fluor 546 (Invitrogen, A10036) and goat anti-rabbit Atto 647N (Sigma-Aldrich, 40,839) were used for the primary antibodies made in mice and rabbits, respectively. The slides were rinsed with PBS for 5 min three times while being kept in the dark, and then coverslips were mounted using Fluoroshield. Unexpectedly, we found that Sox2 staining was sometimes unsuccessful. We are not sure of the reason for this because consecutive sections on the same slide (so from the same embryo and treated the same) could have successful or unsuccessful Sox2 staining. This means that some of our figure panels contain Sox2 stain while others do not.

### Confocal microscopy and data analysis

Brightfield and confocal z-stacks were taken using a Zeiss LSM 900 Airyscan 2 microscope within the Cambridge Advanced Imaging Centre and a Zeiss Cell Discoverer 7 Airyscan 2 microscope within the Manchester Bioimaging facility; ×10 and ×20 objectives were used with a numerical aperture of 0.9–0.95. Images were processed to generate maximum intensity projections using Fiji (Schindelin et al., 2012). Nuclei counts and tail length measurements were carried out manually using Fiji (Schindelin et al., 2012). There was a clear difference between nuclei brightly expressing Sox2 and those only dimly expressing Sox2 (the mean Sox2 intensity ratio of bright vs. dim nuclei in all embryos analyzed was 1.66, with a standard deviation of 0.20). Therefore, these were also categorized manually. Only embryos in which the location of the NEC could be clearly defined in relation to the posterior tip of the tailbud were used for analysis. Statistical analysis was carried out in GraphPad Prism version 11.0.1 for macOS. Specific tests are detailed in figure legends.

## Results

### There is no mesencymal tailbud in classes only exhibiting primary neurulation

The Deuterostome clade can be subdivided into Chordata and Ambulacraria, the latter of which can be further divided into Echinodermata and Hemichordata. Scanning electron microscopy of the Hemichordata collar cord shows it to form by a dorsal folding mechanism along the whole anterior–posterior length, analogous to primary neurulation (Kaul and Stach, 2010; Kaul-Strehlow and Stach, 2013). The Echinoderm nervous system is less similar to that of chordates and is not directly homologous (Kaul and Stach, 2010) because the tubular structure of the nerve cord is thought to have evolved independently from chordates (Märkel and Röser, 1991). However, neuroepithelial thickening and folding have been observed in echinoids, leading to this process being described as “neurulation-like” and suggesting that the formation of hollow nerve cords might be conserved within the Deuterostomes (Märkel and Röser, 1991). Together, this evidence points toward secondary neurulation being a chordate-novel mechanism. To determine which subsets of chordates might exhibit secondary neurulation and which exhibit exclusively primary neurulation, we carried out a review of the existing literature (Figure 1).

**FIGURE 1:**
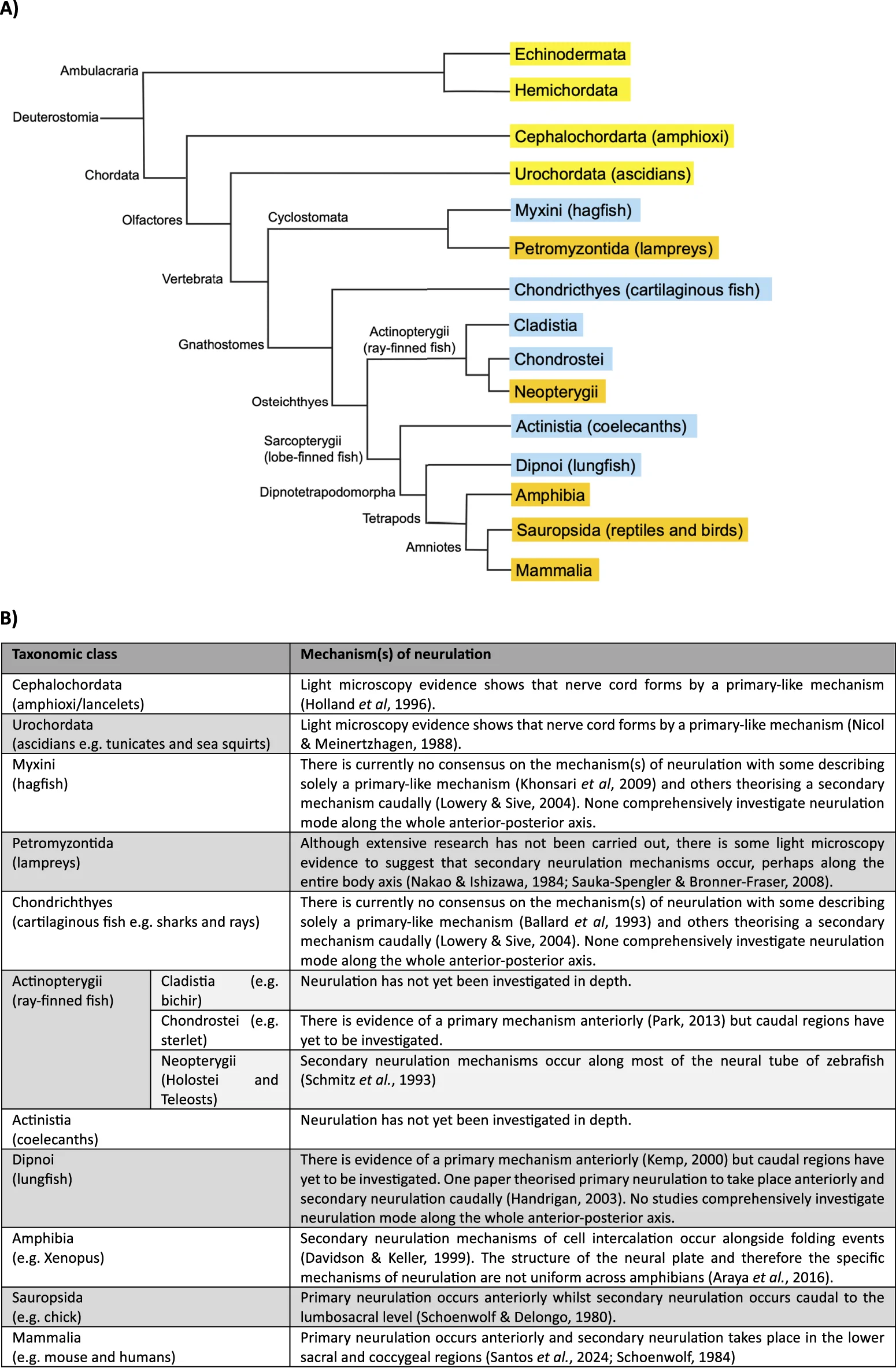
A summary of the currently documented mechanisms of neurulation in the Deuterostomia. **(A)** A phylogenetic tree was constructed using information from Amemiya et al. (2013), Delsuc et al. (2006), Kocot et al. (2018), and Marletaz et al. (2024). Color coding represents the mode of neurulation in each class/subclass. Yellow boxes indicate that primary neurulation forms the entire neural tube. Orange boxes indicate documented incidence of secondary neurulation somewhere along the anterior–posterior length of the neural tube. Blue boxes represent taxonomic classes in which secondary neurulation has not yet been fully investigated. **(B)** Table detailing currently published modes of neurulation. The following references were used to compile this information: Holland et al., 1996; Nicol and Meinertzhagen, 1988; Khonsari et al., 2009; Lowery and Sive, 2004; Nakao and Ishizawa, 1984; Sauka-Spengler and Bronner-Fraser, 2008; Ballard et al., 1993; Park, 2013; Schmitz et al., 1993; Kemp, 2000; Handrigan, 2003; Davidson and Keller, 1999; Araya et al., 2016; Schoenwolf and Delongo, 1980; Santos et al., 2024; Schoenwolf, 1984.

Figure 1 demonstrates that the mechanisms of neurulation have not been well characterized across Chordata clades. However, the only clades in which the documented evidence is suggestive of exclusively primary mechanisms of neurulation are the Cephalochordata and Urochordata. Unlike vertebrates, these early-branching chordates do not have a mesenchymal tailbud, and, in larval stages of development, tail extension occurs via cell rearrangement and tissue elongation of already differentiated structures (Carvalho et al., 2021; Katsuyama et al., 1999; Nicol and Meinertzhagen, 1988; Schubert et al., 2001). Amphioxus tail elongation continues into larval stages at a slow pace (Mansfield et al., 2015). Whilst the precise cellular mechanisms involved in this later tail elongation are still unknown, the lack of a mesenchymal tailbud component would preclude secondary neurulation from occurring.

There is sporadic evidence of secondary neurulation within the vertebrates in species from within the Petromyzontida and Neopterygii subclasses, as well as in tetrapods. However, most chordates have not been sufficiently researched to determine whether secondary neurulation mechanisms exist in these lineages.

Together, this literature review illustrated that, in taxonomic classes in which organisms only exhibit primary neurulation, tail extension appears to occur in the absence of a proliferating population of mesenchymal cells in the tailbud. This raises the hypothesis that secondary neurulation mechanisms may have arisen in the Vertebrata subphylum to facilitate the incorporation of mesenchymal tailbud cells into the already polarized neural tube and that all chordates from vertebrates onward might undergo secondary neurulation in at least part of their body axis.

### Onset of secondary neurulation correlates with tailbud extension

To further explore this hypothesis, we compared the documented developmental timings of tailbud extension and secondary neurulation within representative species from the vertebrate clades known to undergo secondary neurulation. Using this information, we statistically correlated the developmental timings of secondary neurulation and tailbud initiation (Figure 2).

**FIGURE 2:**
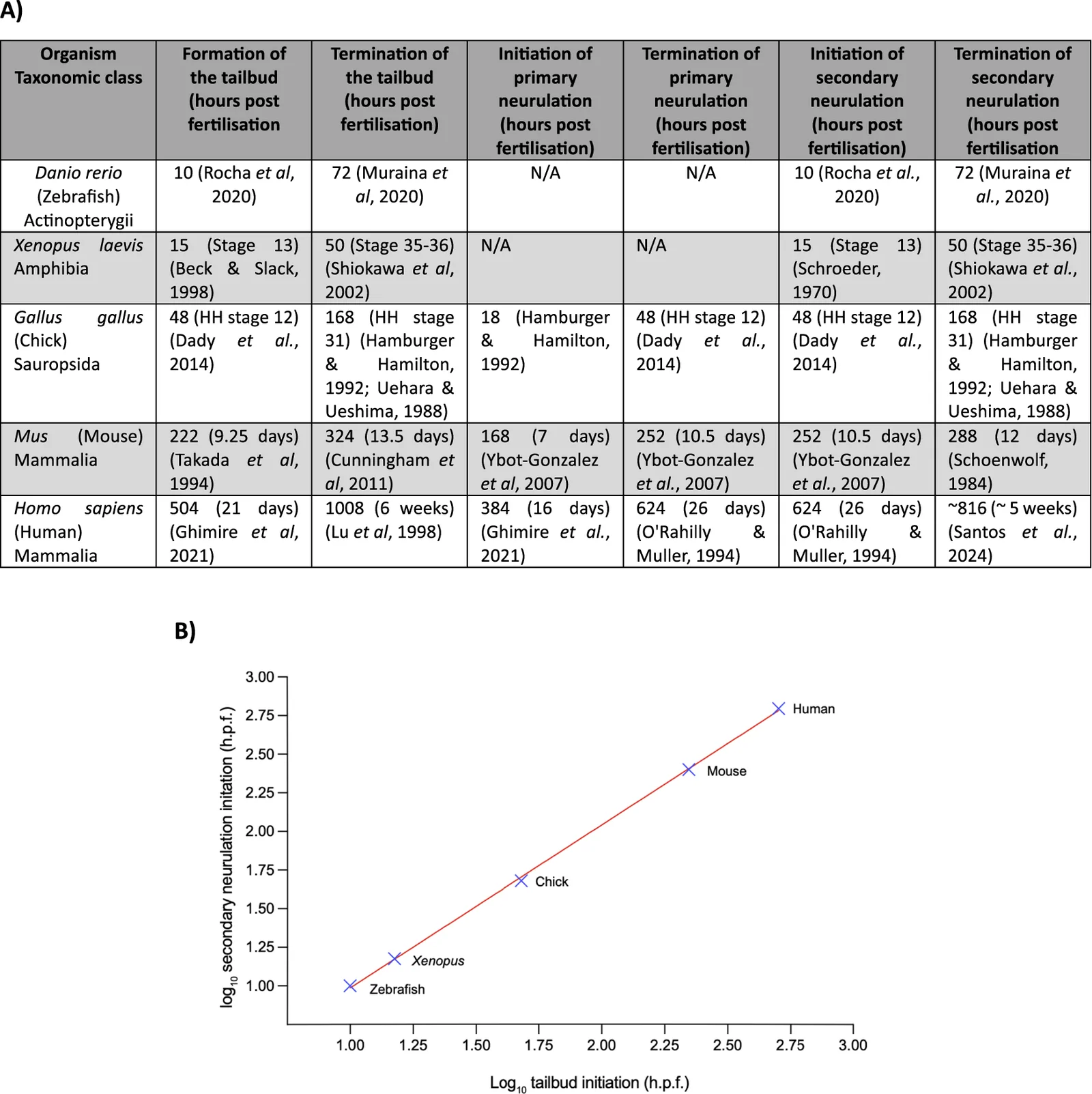
Comparison of the timings of tailbud extension and secondary neurulation. **(A)** Table of developmental timings for primary and secondary neurulation. In organisms displaying both types of neurulation, the literature is consistent in defining that secondary neurulation begins upon closure of the posterior neuropore, which also marks the end of primary neurulation. In zebrafish and *Xenopus* that display secondary-like neurulation along most of their anterior–posterior axis, the start of secondary neurulation was taken as the point at which the neural plate has formed, immediately before the onset of midline intercalation to form the neural keel. If the literature’s consensus on timing was a range, the mean was taken. The following references were used to compile this information: Rocha et al., 2020; Muraina et al., 2020; Beck and Slack, 1998; Shiokawa et al., 2002; Schroeder, 1970; Dady et al., 2014; Hamburger and Hamilton, 1992; Uehara and Ueshima, 1988; Takada et al., 1994; Cunningham et al., 2011; Ybot-Gonzalez et al., 2007; Schoenwolf, 1984; Ghimire et al., 2021; Lu et al., 1998; Ghimire et al., 2021, O’Rahilly and Muller, 1994; Santos et al., 2024. **(B)** Correlation between the time of tailbud initiation and onset of secondary neurulation. Pearson’s correlation coefficient is 0.9998, *p* < 0.0001.

The very strong positive correlation demonstrates that the onset of secondary neurulation coincides with that of tailbud extension. To test this correlation further, we also checked the relative timings of tailbud extension and secondary neurulation for another experimental teleost, the medaka fish. The developmental time of segmentation (and therefore tailbud elongation initiation) in medaka is approximately stage 18–19 (Iwamatsu, 2004). Secondary neurulation in medaka, such as other teleosts, occurs along most of the body axis. The initial polarization of cells (i.e., MET) is initiated at approximately stage 19 (Miyayama and Fujimoto, 1977), therefore closely correlating with the initiation of tailbud extension.

Whilst the correlation between the timing of secondary neurulation and tailbud extension onset might be expected for amniote species, which undergo primary neurulation followed by secondary neurulation when their tails extend, the fact that this correlation also holds true for species such as zebrafish, *Xenopus*, and medaka, in which secondary-like neurulation occurs along the majority of their body axis (not only their tail), gives weight to the argument that secondary neurulation evolved to enable neural tube extension during mesenchymal tailbud extension.

Together, data from the literature are consistent with the hypothesis that secondary neurulation evolved in the vertebrate subphylum to enable neural tube extension during mesenchymal tailbud elongation.

### Secondary-like neurulation is likely present across the Actinopterygii class

To test this theory, we investigated the modality of neurulation within the Actinopterygii class of ray-finned fish (Figure 3).

**FIGURE 3:**
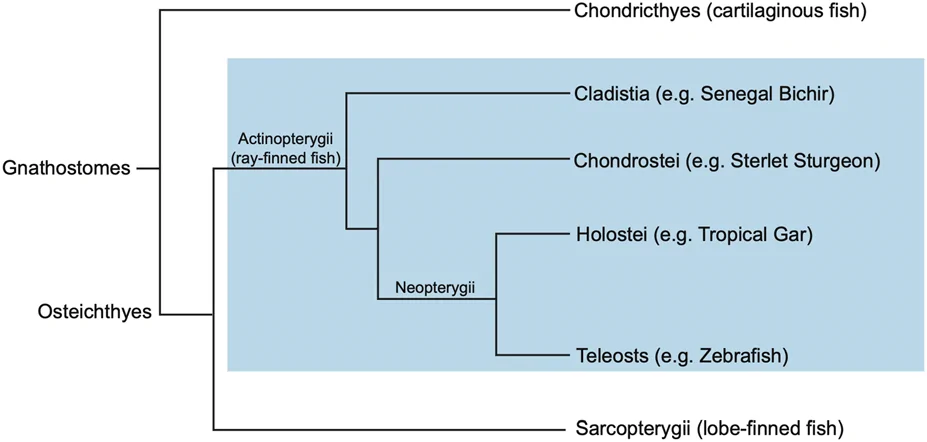
Simplified phylogenetic tree of Actinopterygii. The blue box indicates Actinopterygii. This figure was based on information from Faircloth et al. (2013).

It has been well-established that most of the anterior–posterior extent of the Teleost neural tube forms by a secondary-like mechanism, as exemplified by the zebrafish (Geldmacher-Voss et al., 2003; Schmitz et al., 1993). However, the mechanisms at work in the remaining clades of Actinopterygii are far less clear. Formation of the neural tube in Cladistia and Holostei has not been investigated in depth. In Chondrostei, one light microscopy study identified the neural tube of the Siberian sturgeon (*Acipenser baerii*) to form by a folding-like mechanism between stages 16–18 in the region of the head, resembling primary neurulation (Park et al., 2013). However, only anterior regions were investigated. Given our understanding of neural tube formation in the zebrafish, we might predict other animals belonging to the same Actinopterygii class to also exhibit secondary-like neurulation. We therefore investigated morphological changes during neurulation within the Actinopterygii. First, we digitally re-sliced and analyzed micro-computerized tomography (CT) scans of one species for each of Cladistia (the Senegal bichir), Chondrostei (the sterlet sturgeon), and Holostei (the tropical gar) (Figure 4).

**FIGURE 4:**
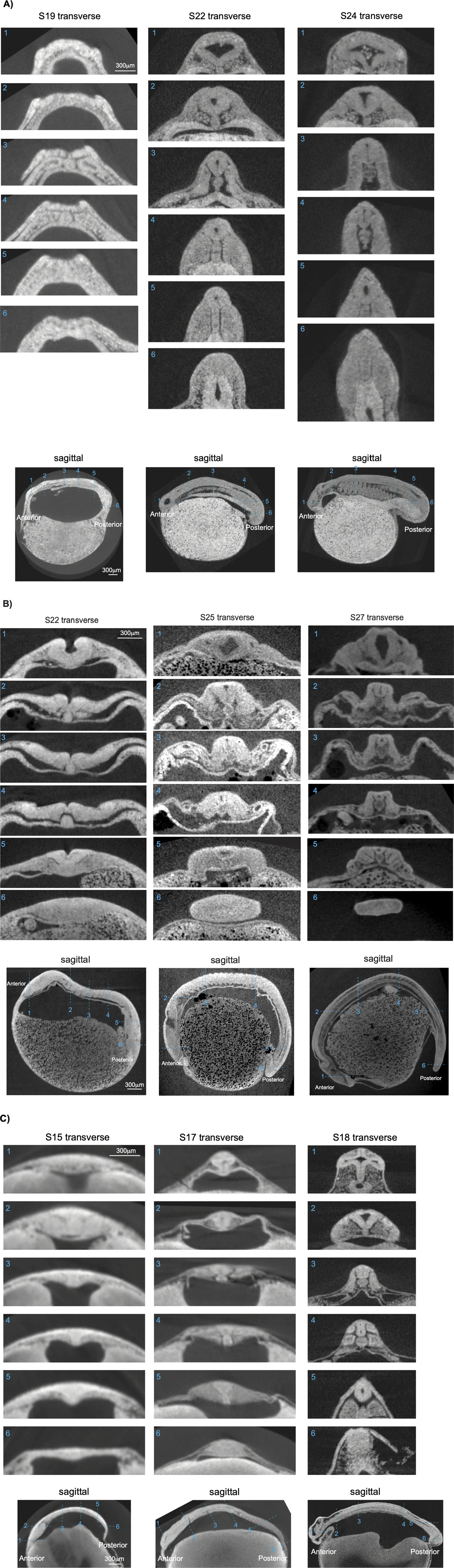
Micro-CT scans for **(A)** bichir, **(B)** sterlet, and **(C)** gar embryos, at neural plate, tailbud, and elongating tailbud stages. Scans were digitally sliced in the transverse orientation along the anterior–posterior axis. The locations of specific numbered sections are indicated on sagittal sections at the bottom of each panel. Scale bars for each panel indicate approximate scale for all embryos of each species.

The transition from an open neural plate to a closed neural tube over the course of development demonstrated that both Senegal bichir (Cladistia) and sterlet sturgeon (Chondrostei) embryos exhibit primary neurulation in anterior regions (Figures 4A,B). The tailbud stage of these embryos was made up of a pronounced, undifferentiated region (Figures 4A,B), which is likely to represent a mesenchymal neural rod and is therefore suggestive that secondary mechanisms of neurulation might occur in extending posterior regions. The transition from a solid neural keel to a polarized neural rod and then to an open neural tube was seen over the course of tropical gar (Holostei) development (Figure 4C). This demonstrated that Neopterygii fish, including the previously characterized teleosts, neurulate via secondary-like mechanisms along most of their axis. Together, these data are highly suggestive that all species within Actinopterygii undergo secondary neurulation mechanisms along at least some of their axis. However, higher resolution images are required to confirm the presence of mesenchymal, neural-fated cells in the tailbud of Cladistia and Chondrostei embryos.

### The sterlet embryo neural tube is formed via primary mechanisms until the neuroenteric canal

To further investigate the mechanisms of neurulation at the tailbud in basal Actinopterygii fish at cellular resolution, we fixed and sectioned sterlet sturgeon (Chondrostei) embryos from the neural plate stage (S20) to the stage of early tail elongation (S27). Transverse sections demonstrated that, such as other documented vertebrates, the sterlet neural tube comprises a Sox2-positive pseudostratified neural epithelium, with zona-occludens-positive apical-lateral junctions lining the neural tube (examples indicated by open arrowheads in Figures 5Bii,iii,Ci).

**FIGURE 5:**
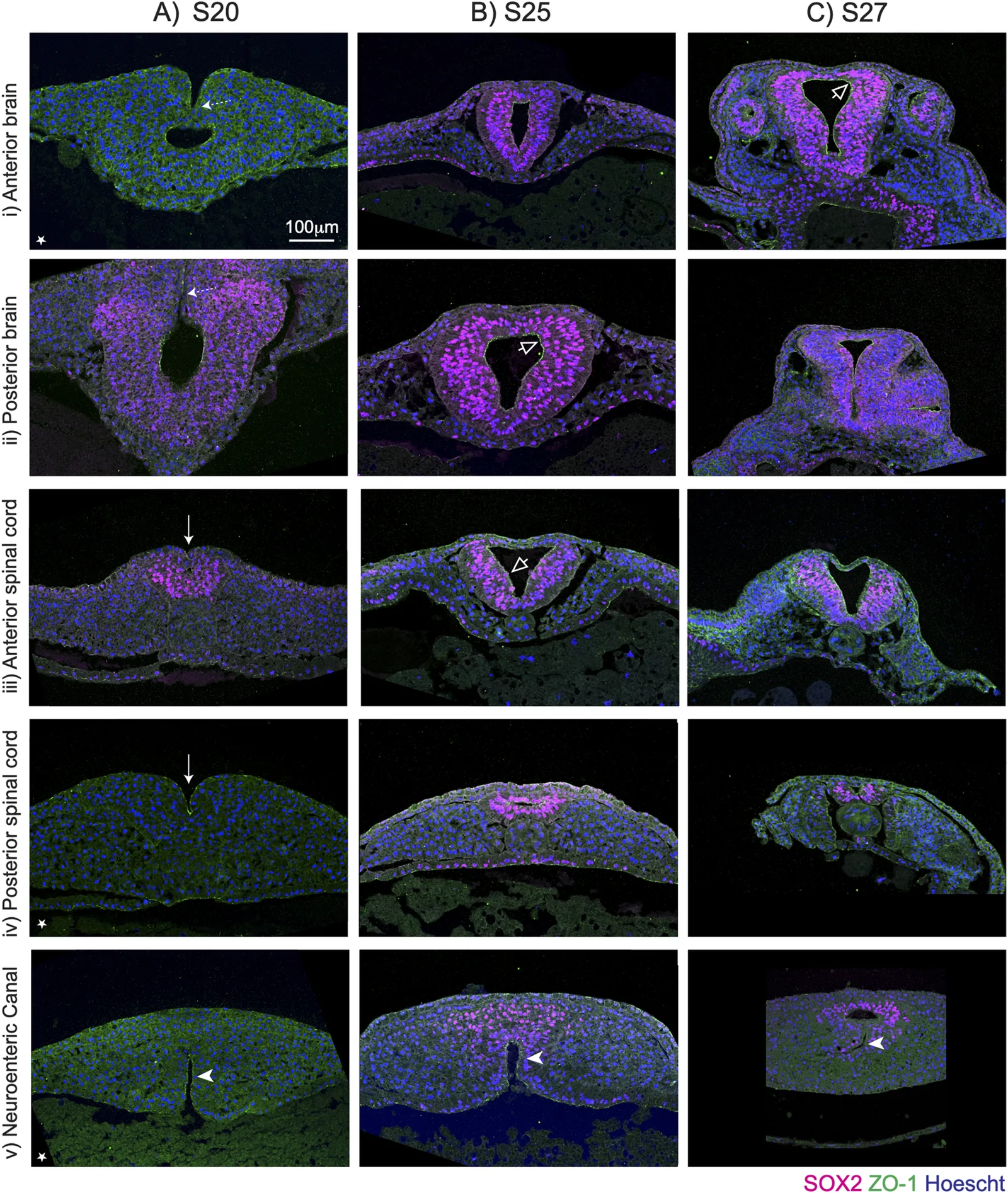
Transverse sections along the anterior–posterior extent of the neural tube of sterlet embryos. Some sections were not stained for Sox2 (white star). Representative images of a **(A)** Stage 20 embryo, **(B)** Stage 25 embryo, and **(C)** Stage 27 embryo. Solid arrows indicate the neural groove. Dashed arrows indicate fusing neural folds. A closing neural plate was seen in 4/4 embryos at S20. Solid arrowheads indicate the neuroenteric canal. Open arrowheads indicate examples of ZO-1-labeled apical-lateral junctions lining the neural tube lumen.

Consistent with microCT scans (Figure 4B), images from S20 embryos demonstrated the presence of a neural groove (arrows) or fusing neural plate (dotted arrows) along most of the anterior–posterior axis (Figures 5Ai–iv). By S25 and S27, the neural plate had fused into a sealed neural tube. This confirmed that most of the anterior–posterior extent of the sterlet neural tube is formed via primary neurulation. At all three stages of development, the more posterior sections illustrated the presence of a neuroenteric canal (NEC, arrowhead in Figures 5Av,Bv,Cv). This is a transient structure connecting the neural tube with the anus (gut tube) and marks the posterior end of the neural tube (Gont et al., 1993).

### Proliferative, Sox2-positive tailbud cells intercalate into the extending neural tube of sterlet embryos

To understand what mechanisms of neurulation occurred within the extending tailbud, we next analyzed sagittal sections to investigate the identity and orientation of cells posterior to the NEC in the tailbud regions (Figure 6). Tail elongation had initiated by S26 (Figure 6B), consistent with the previous description of sturgeon development (Dettlaff et al., 1993). At neural plate stage (S20), Sox2-positive nuclei were visible in the neural plate along the dorsal side of the tail (Figure 6B star), as well as in a small population more ventrally that marked the position of the future NEC (Figure 6B arrow). By early tailbud stage (S25), the NEC was clearly visible, and some Sox2-positive nuclei were present posterior to this structure, in the tailbud regions (Figure 6B arrow), but not at the most posterior regions of the tailbud.

**FIGURE 6:**
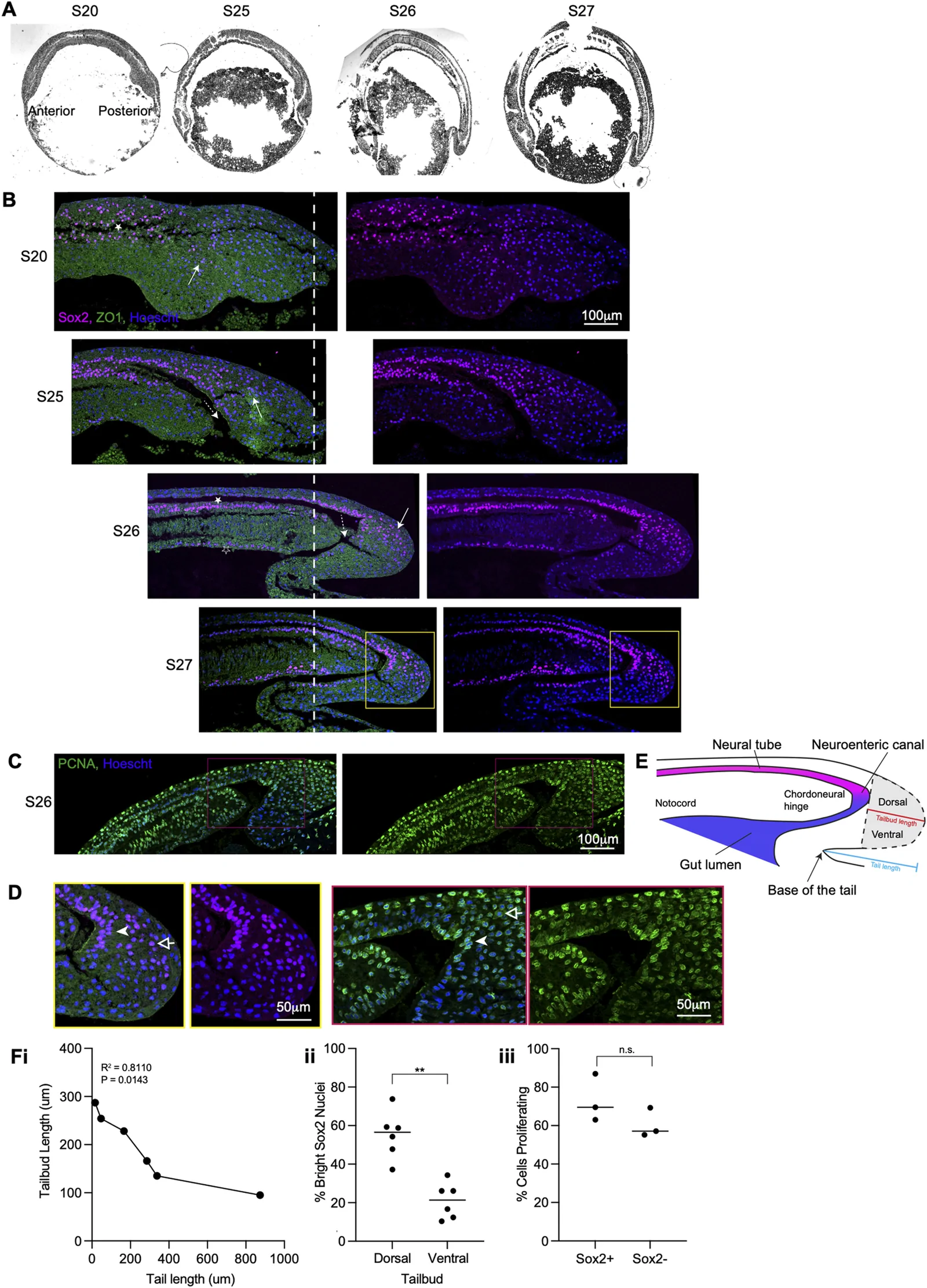
Sagittal sections through the middle of the tailbud of sterlet embryos. **(A)** Brightfield images of whole embryo sections. **(B)** Tailbud sections. White dotted line indicates the position at which the base of the tail joins to the rest of the embryo. S20: Neural plate stage showing Sox2-positive nuclei in the neural plate (star) and at the future NEC (arrow). S25: Tailbud initiation stage showing some Sox2-positive nuclei posterior to NEC (arrow). S26 and S27: Early tailbud extension stages showing Sox2-positive nuclei in the polarized neural tube (star), posterior tailbud (arrow), and gut wall (open star). Different orientations of the NEC at S25 and S26 are indicated by dotted arrows. **(C)** Tailbud sections from extending tailbud, stage S26. Proliferative nuclei were identified by PCNA at the nuclear envelope or as speckled staining inside the nucleus (S-phase) or by PCNA in the cytoplasm (M-phase). Interphase nuclei were identified by uniform staining within the nucleus (G1 and G2 phases). **(D)** Zoomed-in regions from **(B)** and **(C)**, indicating polarized cells at the posterior edge of the NEC. Arrowheads indicate polarized nuclei close to the NEC. Open arrowheads indicate rounded, mesenchymal nuclei distal to the NEC. **(E)** Schematic of tailbud anatomy. Dorsal and ventral tailbud regions used for analysis are indicated in grey. Tail and tailbud length regions for measurement are indicated by the blue and red lines, respectively. **(F)** Quantifications from embryos of stages S25–S28: i) Distance from the base of the tail (white dotted line in B) to the tip of the tail (tail length) plotted against distance from the posterior edge of the NEC to the tip of the tail (tailbud length). Pearson’s analysis demonstrated a significant negative correlation between tail length and tailbud length. ii) Percentage of all nuclei in the dorsal and ventral regions of the tailbud that expressed Sox2 brightly: 55% of dorsal nuclei expressed Sox2 brightly compared with 21% of ventral nuclei (*p* = 0.0026, paired t-test). iii) Percentage of all bright Sox2 nuclei and dim/absent Sox2 nuclei that were proliferating: 73% of bright Sox2 nuclei were proliferative, compared with 61% of dim/absent Sox2 nuclei. However, this small difference was not significant (*p* = 0.0694, paired t-test).

Similar to observations in other vertebrate tailbuds (Sambasivan and Steventon, 2020), by early tailbud extension stages (S26–S27), brightly expressing Sox2 nuclei (more likely to be neural-fated) were clearly localized to the dorsal half of the tailbud, extending to posterior regions (Figure 6B arrow and Fii). Sox2-positive nuclei were present along the gut (Figure 6B open star), as well as in the tailbud and lining the polarized neural tube (star). These were likely enteric nervous system progenitors or the dorsal epithelia of the cloaca that generate the anorectal and urogenital systems (Heanue and Pachnis, 2011; Olivera-Martinez et al., 2012; Row et al., 2016; Runck et al., 2014).

The elongated morphology of Sox2-positive nuclei lining the dorsal side of the posterior NEC demonstrated the polarized nature of these epithelial cells, and these were also highly proliferative (Figure 6D, arrowheads). In contrast, the tailbud nuclei further posterior to the NEC were more rounded in nature, suggesting a mesenchymal state (Figure 6D, open arrowheads). Although not possible to definitively prove without live imaging (which is not technically feasible), the orientation of cell nuclei near the NEC was suggestive of a mesenchymal to epithelial transition and intercalation of tailbud cells into the dorsal half of the polarized NEC.

Both pre-NEC polarized epithelial neural tube and chordoneural hinge cells, and post-NEC unpolarized mesenchymal tailbud cells were proliferative (Figure 6C). This demonstrates that both anterior and posterior mechanisms of neural tube elongation are likely to coexist. There was no difference in proliferation between bright Sox2-positive nuclei (more likely to be neural-fated) and dim/absent Sox2 nuclei (more likely to be mesoderm-fated) (Figures 6C,Fiii). This suggests that the rate of proliferation was relatively uniform throughout the tailbud and between neural and mesoderm-fated cells. Plotting individual tail lengths against tailbud length demonstrated that as embryos develop and extend their tails, the mesenchymal tailbud size is reduced (Figure 6Fi). This might suggest that cell intercalation into differentiated tail structures is faster than cell proliferation of mesenchymal cells within the tailbud. It is not possible from these data to determine the relative contributions of anterior vs. posterior mediated tail elongation or of proliferation vs. cell rearrangement/tissue expansion as mechanisms of elongation. However, these data do demonstrate that Sox2-positive mesenchymal cells proliferate at the onset of tail elongation and intercalate with already polarized neuroepithelial cells via secondary mechanisms of neurulation.

### Tailbud neurulation in sterlet embryos occurs via intercalation of neural progenitor cells, not by *de novo* lumen formation

To determine whether mesenchymal tailbud cells undergo *de novo* polarization and lumenogenesis (as in chicks and in teleosts) or intercalate into an already polarized epithelium without *de novo* polarization (as in mice and *Xenopus*), we studied the localization of the tight junction marker zonula occludens 1 (ZO-1). ZO-1 is a scaffolding protein, found to localize at the apical membrane initiation site during *de novo* polarization in multiple models of lumen formation, as well as at apical-lateral junctions in fully epithelial cells (Buckley and St Johnston, 2022).

Serial transverse sections were taken of the posterior regions from tailbud initiating (S25) and tailbud elongating (S26 and S27) sterlet embryos (Figure 7). These demonstrated the resolution of the neural tube lumen into the neuroenteric canal and then the mesenchymal tailbud. From anterior to posterior S25 sections (Figure 7A), the dorsally located neural tube (star) gradually transitions into the more ventral NEC (dotted arrow) and then the ventrally located gut (open star). There is no area of enhanced ZO-1 staining in the tailbud. In S26 and S27 sections (Figures 7B,C), the neural tube, NEC, and gut are all visible at the same axial level. This is because, due to the elongation of the chordoneural hinge from S26 onward, the morphology of the NEC changes from S25 to S26. At S25, the NEC is present as a ventrally sloping canal along the anterior–posterior axis, while at S26, it has a much more upright orientation (dotted arrows in Figure 6B). The S26/27 NEC lumen then gradually reduces in size at the posterior side of the NEC, until a pronounced region of ZO-1 polarization, marking the end of the NEC (Figure 7, arrowheads). Similar to previous observations in the mouse tailbud (Schoenwolf, 1984), this is surrounded by an accumulation of nuclei at the most posterior slice, suggesting the interdigitation of tailbud cells into the polarized neuroepithelium (Figure 7, arrows). At all three stages of development, there was no evidence of ZO-1 polarization beyond the posterior edge of the NEC, suggesting that *de novo* polarization and the formation of initially separate lumens (as in chicks) does not occur in sterlet embryos. Together with Figure 6, these data strongly suggest that, as the tailbud extends, the proliferating neural progenitor cells from the tailbud undergo MET and intercalate into the existing neural tube epithelium. This coordinates neural tube and tailbud elongation.

**FIGURE 7:**
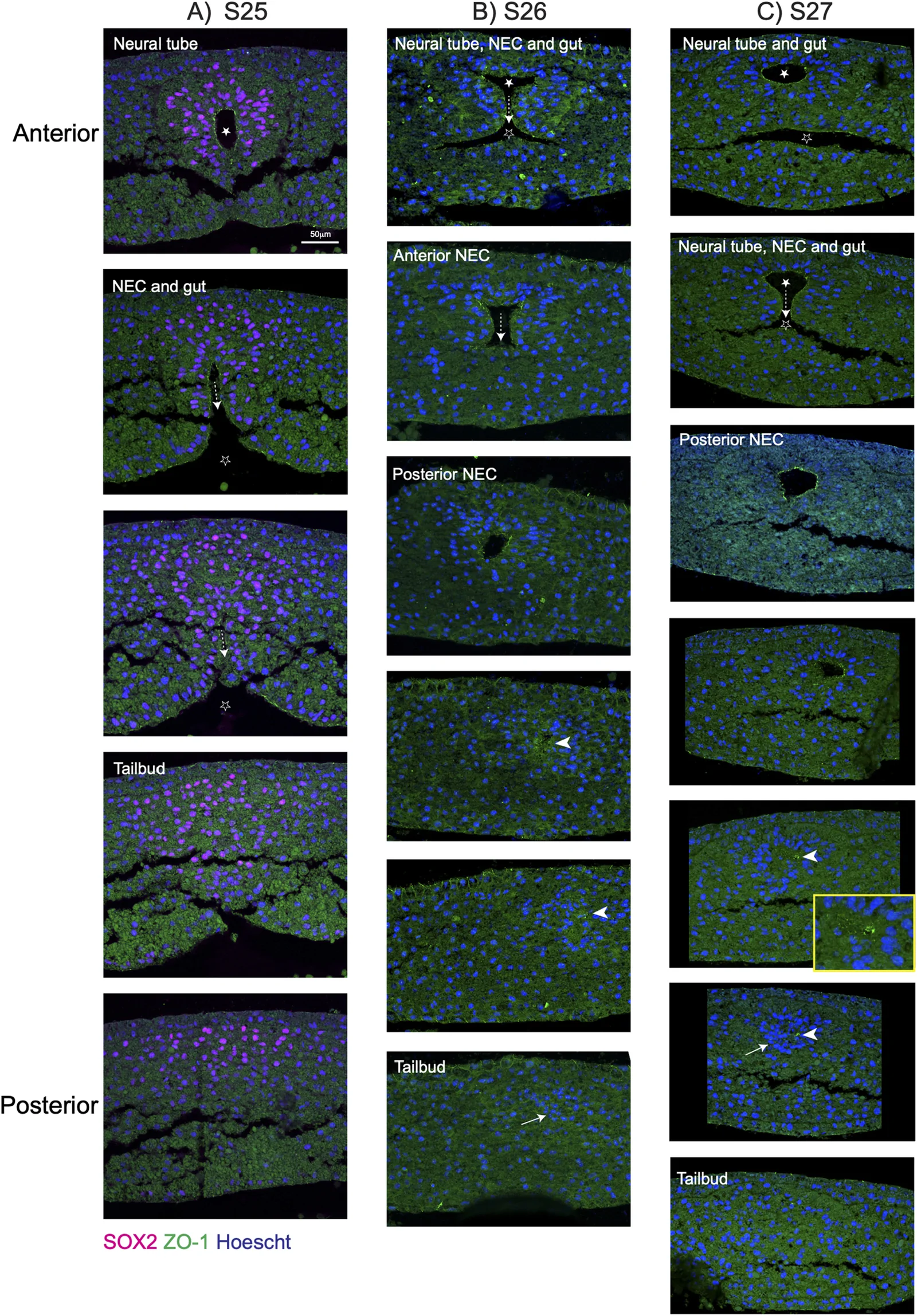
Transverse sections along the neuroenteric canal and tailbud of **(A)** S25, **(B)** S26, and **(C)** S27 sterlet embryos. Sox2 was stained in S25 sections but not in S26 or S27 sections. Stars indicate neural tube lumens. Dotted arrows indicate the orientation of the NEC. Open stars indicate gut lumens. Arrowheads indicate the end of the lumen, lined with ZO-1-positive apical-lateral junctions. An inset image shows an enlargement of this junctional region in S27. Arrows indicate an accumulation of nuclei at the posterior-most end wall of the NEC lumen.

## Discussion

Here we conclude that secondary neurulation mechanisms are likely present throughout the Vertebrata clade, evolving to enable the incorporation of cells generated within a mesenchymal tailbud into an already polarized posterior spinal cord. However, even among already well-documented cases of secondary neurulation within this clade, multiple overlapping cellular mechanisms exist (see Introduction). For example, while avians such as chicks make multiple lumens in the tailbud via *de novo* polarization, which then fuse with the posterior spinal cord via cell intercalation, the prevalence of *de novo* lumenogenesis as a secondary neurulation mechanism within the tailbud of non-avian amniotes is not clear. Our study demonstrates that, while cell intercalation is present within Chondrostei fish, *de novo* lumenogenesis is not. Conversely, our study suggests that all Neopterygii fish (not only the teleosts) are likely to neurulate via cell intercalation and *de novo* polarization along most of their anterior–posterior axis. The reason for the different adoption of secondary mechanisms even within the same Actinopterygii class remains uninvestigated.

We propose that the common cellular requirement during secondary neurulation is the need for an initially solid mesenchymal population of cells to generate a hollow polarized epithelial tube. Therefore, variations in the specific mechanisms of neurulation used between species and even between different anterior–posterior extents of the neural tube within the same organism likely reflect differences in relative rates of body axis elongation, mesenchymal cell proliferation, and MET. These differences would result in different geometrical constraints, resulting in variations of the overall modality of deployment for the MET that is used to generate a polarized tube.

For example, for species in which posterior mesenchymal cells proliferate rapidly within the tailbud, larger tailbuds might be produced. Cells undergoing MET within larger tailbuds would initially be physically distal from the posterior end of the already polarized tube, which might result in *de novo* polarization into multiple lumens that later coalesce. For example, the chick tailbud (which forms multiple lumens) is large (∼400–500 µm long from HH12–15, for example, images in Guillot et al. (2021)), and this large size is maintained during tailbud extension due to proliferation of NMPs (Benazeraf et al., 2017). This hypothesis is supported by strong *in vitro* evidence that demonstrates that the number of lumens emerging in polarizing neural rosettes increased within larger areas of tissue, whilst higher tissue confinement led to single lumens (Knight et al., 2018). Conversely, in species in which posterior mesenchymal cells proliferate less or in which the process of MET is faster in relation to this proliferation, cells undergoing MET would be more proximal to the posterior end of the already polarized tube and could intercalate directly, without an intermediate *de novo* polarization into separate lumens. The size of the sterlet tailbud (which forms a single lumen) decreases as the tail length extends (Figure 6F), and cells directly intercalate into the existing NEC (Figure 6D), supporting this theory.

However, the overall size of the tailbud cannot be the only parameter at play because secondary neurulation in rodents only form a single medullary lumen (Schoenwolf, 1984), despite the large increases in tailbud volume due to high rates of NMP proliferation (Sambasivan and Steventon, 2020). It would therefore be interesting to compare whether the rates of MET differ between chick and mouse. It is also likely that the overall geometric shape and cell density of the tailbud play a role in when and where MET occurs.

Similarly, the differences between species that undergo secondary neurulation solely at their tailbud vs. those that adopt these mechanisms along most of their anterior–posterior axis are likely to be related to the timing of tail elongation in relation to MET. For example, zebrafish rapidly elongate their tails from very early stages of development (10 h post fertilization). At this stage, many neuroepithelial progenitor cells have not yet completed MET (e.g., in the hindbrain (Tawk et al., 2007)). Therefore, only secondary neurulation mechanisms of cell intercalation and *de novo* polarization would enable tail elongation and MET to occur at the same time. The more fluid nature of these cells may also facilitate faster body axis elongation in this rapidly developing species. In contrast, sterlet embryos only form their tailbud at stage 25, which is approximately 70–80 h post fertilization. This is long after the MET of neuroepithelial cells, which form a polarized neural plate by stage 19 (∼32–36 h post fertilization) (Dettlaff et al., 1993) and have therefore finished primary neurulation by the time the tailbud is formed. This slower rate of development might therefore result in the restriction of secondary neurulation mechanisms to tailbud regions. Future studies should investigate the relative timings and rates of body axis elongation, mesenchymal cell proliferation, and cell polarization (MET) between species and compare them to the specific secondary neurulation mechanisms used.

## Conclusion

A re-evaluation of current literature demonstrated that there is no mesenchymal tailbud in classes only exhibiting primary neurulation and that the mechanisms of neurulation have not been fully investigated for most chordates. It also demonstrated that the developmental onset of secondary neurulation coincides with the start of posterior tailbud extension in all documented cases. Next, we further investigated the mechanisms of neurulation within Actinopterygii fish, within which only teleosts such as zebrafish have previously been shown to neurulate via a secondary-like mechanism. Micro-CT scan analysis demonstrated the presence of secondary-like neurulation all along the body axis in Holostei. It also demonstrated the presence of primary neurulation along most of the body axis in Chondrostei and Cladistia, except within an undifferentiated tailbud region. To investigate the mechanisms of neurulation within this tailbud region, we carried out immunohistochemistry analysis of Chondrostei sterlet sturgeon (*Acipenser ruthenus*) embryo sections. This confirmed that the anterior portion of the sterlet neural tube forms by primary neurulation until a neuroenteric canal. Posterior to the NEC, a mesenchymal population of Sox2-positive cells is present along the dorsal half of the tailbud. There was no evidence of *de novo* cavitation within the tailbud. Instead, Sox2-positive cells proliferate at tailbud extension stages and intercalate into the extending NEC.

Together, we conclude that secondary mechanisms of neurulation arose within the Vertebrata to enable mesenchymal tailbud extension. We advocate for the investigation of the cellular mechanisms of neurulation in other chordate species to better understand how variations in secondary-like mechanisms (e.g., whole body-axis vs. tailbud secondary neurulation, or intercalation only vs. *de novo* cavitation and intercalation) arose. We propose that these variations are likely to be the consequence of different geometrical constraints due to differences in relative rates of body axis elongation, mesenchymal cell proliferation, and cell polarization.

## Data Availability

The original contributions presented in the study are included in the article, further inquiries can be directed to the corresponding author.
